# Regional Tear Film Quality Changes Across Eye Gaze Directions Revealed by Spatial Mapping and Threshold Sensitivity Analysis

**DOI:** 10.3390/jcm15155921

**Published:** 2026-07-29

**Authors:** Wen-Pin Lin, Rowan Abass, Wei-Ren Lin, Richard Wu, Arwa Fathy, Ahmed Abass

**Affiliations:** 1 Department of Optometry, Mackay Medical University, New Taipei 252, Taiwan; 2 Research and Development Centre, Brighten Optix Corporation, Taipei 111, Taiwan; 3 Department of Medicine, University of Cambridge, Cambridge CB2 0SP, UK; 4 Department of Optometry, University of Kang Ning, Taipei 114, Taiwan; 5 College of Optometry, Pacific University, Forest Grove, OR 97116, USA; 6 Department of Engineering, University of Cambridge, Cambridge CB2 1PZ, UK; 7 Department of Materials, Design and Manufacturing Engineering, University of Liverpool, Liverpool L69 3GH, UK

**Keywords:** tear film, gaze direction, Medmont, ocular surface, dry eye, tear-film quality, spatial mapping, threshold analysis

## Abstract

**Objectives**: This study aimed to determine whether eccentric gaze produces a regional, gaze-aligned redistribution of tear-film quality that is more apparent in spatial analysis than in whole-map summary measures. **Methods**: This was a secondary analysis of a previously acquired Medmont Meridia composite-topography dataset from 38 healthy participants, using only the Medmont data. Tear-film quality maps from the ahead, right, left, up, and down gaze were interpolated onto a common grid, aligned to a shared reference frame, and analysed using descriptive global measures, regional spatial mapping, and a threshold-based directional sector analysis. The threshold analysis used an operational cut-off of 0.35 to classify good-quality pixels, with lower values indicating better tear-film quality. Robustness was assessed by repeating the same analysis across nearby cut-offs from 0.30 to 0.40. As Placido-based topography measures reflection from the tear layer rather than the ocular surface itself, and as ocular rotation changes lid position and exposed ocular surface area, aligned comparisons were interpreted as comparisons of the exposed tear-film pattern rather than exact point-for-point anatomical tracking. **Results**: Global tear-film summary measures showed only modest variation with gaze direction, whereas spatial mapping revealed systematic regional redistribution of tear-film quality across eccentric gaze conditions. At the 0.35 operational threshold, the gaze-aligned good-pixel index was positive for right gaze (0.297 ± 0.200), left gaze (0.213 ± 0.174), up gaze (0.204 ± 0.219), and down gaze (0.308 ± 0.260). All four indices remained greater than zero after Holm correction (all pHolm < 0.001), and all remained greater than the matched ahead-gaze sector indices (all pHolm < 0.001). Threshold sensitivity analysis across cut-offs from 0.30 to 0.40 showed that the positive directional pattern was preserved for all gaze directions. **Conclusions**: Tear-film quality varies regionally with gaze direction, a pattern that was clearer in spatial maps and sector indices than in global summaries. Straight-ahead measurements may therefore provide an incomplete account of tear-film behaviour during eccentric gaze. As the analysis involved healthy participants and did not assess symptoms or diagnostic performance, the clinical relevance of this pattern requires validation in symptomatic populations.

## 1. Introduction

The tear film is essential for ocular-surface protection, optical quality, and visual comfort. Modern dry-eye research no longer treats tear dysfunction as a simple problem of tear quantity, but instead places loss of tear-film homeostasis and tear-film instability at the centre of disease understanding [1,2]. The tear film is a dynamic structure with behaviour that depends on lid interactions, surface properties, and environmental conditions rather than on tear volume alone [3]. The wider biological importance of the tear film for comfort, infection control, wound healing, debris clearance, and maintenance of optical quality has likewise been reviewed in detail [4,5].

These ideas have clear implications for diagnosis. The TFOS DEWS II Diagnostic Methodology Report identifies tear-film stability as one of the most informative signs in dry-eye assessment and recommends a structured interpretation of symptoms, break-up time, osmolarity, and ocular-surface staining [6]. The TFOS DEWS II Pathophysiology Report further emphasises that tear instability, hyperosmolarity, surface damage, and inflammation interact in a self-perpetuating cycle [7]. Together, these reports frame tear instability as a dynamic surface phenomenon, which raises an obvious but still underexplored question: does tear behaviour change meaningfully when the eyes move away from straight-ahead gaze?

There is already some evidence that gaze direction matters. Pansell, Porsblad, and Abdi showed that tear-film stability changes with vertical gaze position, with poorer stability in upward gaze than in downward gaze, and suggested that changes in palpebral fissure height and exposed ocular surface area were likely contributors [8]. More recently, Covic and colleagues reported that greater palpebral fissure height in primary gaze was associated with poorer tear-film stability in healthy eyes, further supporting the relevance of exposure geometry [9].

Emerging work also suggests that the effect of gaze may be regional rather than global. Ax and colleagues showed that changes in vertical gaze can expose previously lid-covered ocular-surface regions and alter tear-film movement over newly exposed areas [10]. This implies that gaze-related tear changes may involve local redistribution of the exposed tear pattern rather than a simple whole-eye worsening or improvement.

There are also reasons to think that such effects may matter clinically. Blink behaviour is strongly linked to tear stability and ocular-surface exposure. Altered blinking, including incomplete blinking, affects tear parameters and ocular-surface behaviour [11]. Reduced blink rate and incomplete blinks have been reported during computer use and other visually demanding tasks [12,13]. Reviews of digital display use and the ocular surface have likewise linked task-related viewing to dry-eye symptoms and surface alteration [14,15,16]. This suggests that a patient may experience symptoms during eccentric gaze even when a brief straight-ahead assessment appears only mildly abnormal.

Many everyday visual tasks involve sustained non-primary gaze, together with altered blinking and changes in ocular-surface exposure. If gaze-related tear-film changes are spatially local, a single primary-gaze summary may miss the region most affected during the task. This practical gap motivates regional multi-gaze analysis as a research approach, rather than as an established clinical test.

Regional tear-film behaviour is shaped by the interaction of ocular-surface exposure, lid position, tear menisci, blink completeness and lipid-layer continuity [1,17,18,19,20,21,22,23,24,25,26,27,28,29,30,31]. Changes in palpebral fissure height and exposed surface area can alter evaporation, tear thinning and redistribution across the cornea and bulbar conjunctiva [22,23,26,27,28,29,30,31,32,33,34,35,36,37,38]. Table 1 summarises the mechanisms most directly relevant to a regional analysis of gaze.

The present study used a previously reported multi-gaze Medmont dataset [39] to determine whether eccentric gaze produces a regional, gaze-aligned redistribution of tear-film quality that is more apparent in spatial maps and sector indices than in whole-map summary measures. Threshold-sensitivity analysis was used to test the robustness of the directional pattern.

**Table 1:** Region-specific mechanisms directly relevant to gaze-dependent tear-film redistribution.

Tear-Film Function	Central Cornea	Peripheral Cornea and Limbus	Bulbar Conjunctiva Over Sclera
Regional tear-film stability	Mucins, aqueous flow and lipid integrity maintain wettability and delay corneal break-up [1,3,4,6,7,24,25,30,40].	Peripheral break-up contributes to local thinning, redistribution and wider tear-film instability [23,26,27,28,31,41].	Conjunctival fluid and mucins maintain continuity over the exposed surface [24,42,43,44].
Evaporation and lipid-layer continuity	Lipid-layer continuity limits evaporation and stabilises the central tear film [3,30,31,32,45].	Peripheral lipid and meniscus interactions influence evaporation and thinning between blinks [22,23,27,30,31,32,46].	Greater exposed conjunctival area increases evaporative loss and desiccation [22,23,28,34].
Exposure geometry and palpebral fissure	Gaze-related lid position and exposure indirectly affect central stability [8,9,10,22,34,47].	The peripheral cornea and limbus are sensitive to changes in exposure and palpebral fissure height [8,9,10,22,34,43,47].	Exposed bulbar conjunctiva changes with gaze, altering local drying and tear redistribution [22,34,35,43,47].
Tear menisci and blink-mediated redistribution	Central film thickness depends partly on tear menisci and the post-blink film [26,27,29].	Tear menisci replenish the peripheral corneal film after blinking [23,26,27,29].	Meniscus behaviour, blink completeness and conjunctival geometry shape the tear compartment near the lids [26,29,33,37,38].

## 2. Materials and Methods

### 2.1. Study Design and Source Dataset

This was a secondary, within-subject image-analysis study using an existing anonymised dataset comprising 38 healthy participants measured with the Medmont Meridia Composite Topography Wizard under automatic capture conditions [39]. The study involved no new recruitment and was approved by the Institutional Review Board (IRB) in Taiwan (CS1-23215), in accordance with the Declaration of Helsinki.

As the present study was based on an existing paired dataset, a sensitivity-based sample-size justification was used for the current within-subject analyses. With 38 subjects, a two-sided paired comparison at α = 0.05 has 80% power to detect a standardised mean difference of approximately dz = 0.47. For a moderate paired effect size (dz = 0.50), the corresponding power is approximately 85%. Even under a conservative Bonferroni-adjusted threshold for four directional comparisons (α = 0.0125), the available sample remains sufficient to detect an effect of approximately dz = 0.57 with 80% power. These values indicate that the present sample size is adequate for detecting moderate within-subject directional effects.

The inclusion criterion required participants to have no ocular disease other than ametropic refractive error. Exclusion criteria were prior ocular surgery, ocular-surface disease or scarring, connective-tissue disease, pregnancy, and the early postpartum period. Acquisition acceptability was inherited from the source dataset and required successful completion of the Medmont automatic-capture workflow with exportable tear-film quality maps for the required gaze positions. The present analysis introduced no additional manual image selection, relabelling, or exclusion.

The Medmont Meridia software (version 7.2.14) uses a Composite Topography Wizard to acquire a set of five images. When operated in automatic mode, the device first records an image with the subject looking straight ahead. The examiner is then prompted to instruct the participant to look in several additional directions. Once the pupil reaches the predefined displacement from the optical axis, image capture is automatically initiated.

### 2.2. Map Processing and Alignment

Tear-film quality maps from the ahead, right, left, up, and down gaze were automatically exported and processed using custom routines in MATLAB R2026a (The MathWorks Inc., Natick, MA, USA). Gaze-specific maps were interpolated onto a common Cartesian grid and aligned using gaze-dependent translational offsets, allowing the exposed tear-film pattern to be compared in a shared reference frame. This kind of spatial treatment is consistent with earlier work that has combined or extended topographic map information to study ocular-surface shapes beyond a single central view [41,48]. Related topography-based asymmetry work also supports the use of spatially organised map analysis when the clinical signal is expected to be local rather than purely global [47,49].

Right- and left-eye map series were processed and displayed separately in the eye-specific figures. The participant-level inferential summaries reported in Table 2 and Table 3 were used to quantify the directional pattern without fitting a pooled fellow-eye model.

The alignment step enables regional comparisons, but its meaning should be stated carefully. Placido-based topography measures reflection from the tear layer rather than the ocular surface itself, and the source Medmont paper explicitly notes that tear-layer variation during multiple-shot acquisition is unavoidable [39]. In addition, ocular rotation changes lid position and the extent of the exposed ocular surface [10]. For that reason, the present comparisons are interpreted as comparisons of the aligned exposed tear-film pattern rather than exact anatomical tracking of identical surface points across gaze positions. For horizontal gaze, sector assignment was referenced to the subject’s gaze direction rather than the observer-view topography display, so that gaze-aligned and opposite sectors reflected anatomical gaze direction rather than screen orientation.

Global tear-film behaviour was summarised descriptively using whole-map measures. Regional analysis was then performed as the mean maps suggested that gaze changed the spatial distribution of tear-film quality more clearly than a single global average. Ahead gaze served as the principal reference condition. In the analytical hierarchy of the present study, these global measures were descriptive, the regional map comparison was the primary spatial analysis, and the threshold-based sector analysis was the principal subject-level quantitative endpoint.

A directional sector analysis was performed to test whether threshold-defined good-quality regions showed a systematic spatial relationship to gaze direction. As lower tear-film quality values indicated better quality in the exported maps, pixels with values below 0.35 were classified operationally as good-quality pixels. The 0.35 threshold was not treated as a universal clinical boundary. Instead, it was used pragmatically to segment the lower-value, higher-quality portion of the mapped tear-film quality range, as this threshold captured the visually prominent, higher-quality regions while remaining within the observed map range. To reduce the risk of post hoc over-interpretation, the same directional analysis was repeated across nearby thresholds from 0.30 to 0.40 as a formal sensitivity analysis.

### 2.3. Statistical Approach

Global map summaries were reported descriptively. The primary subject-level outcome was the gaze-aligned good-pixel index, calculated as the proportion of pixels below the operational threshold in the gaze-aligned sector minus the corresponding proportion in the opposite sector. At the 0.35 threshold, two-sided one-sample *t*-tests assessed whether each directional index differed from zero, and two-sided paired *t*-tests compared each eccentric-gaze index with its matched ahead-gaze sector index. Results are reported as mean ± standard deviation (SD), 95% confidence interval (CI), t-statistic with 37 degrees of freedom, two-sided p-value, and Cohen’s dz where applicable. Holm correction was applied across the four directional comparisons. The same analysis was repeated for cut-offs from 0.30 to 0.40. Regional significance maps were treated as descriptive pointwise summaries and were not included in the confirmatory family of tests. All 38 participants contributed to each primary comparison. Data processing and statistical analyses were performed in MATLAB R2026a (The MathWorks Inc., Natick, MA, USA).

**Figure 1:**
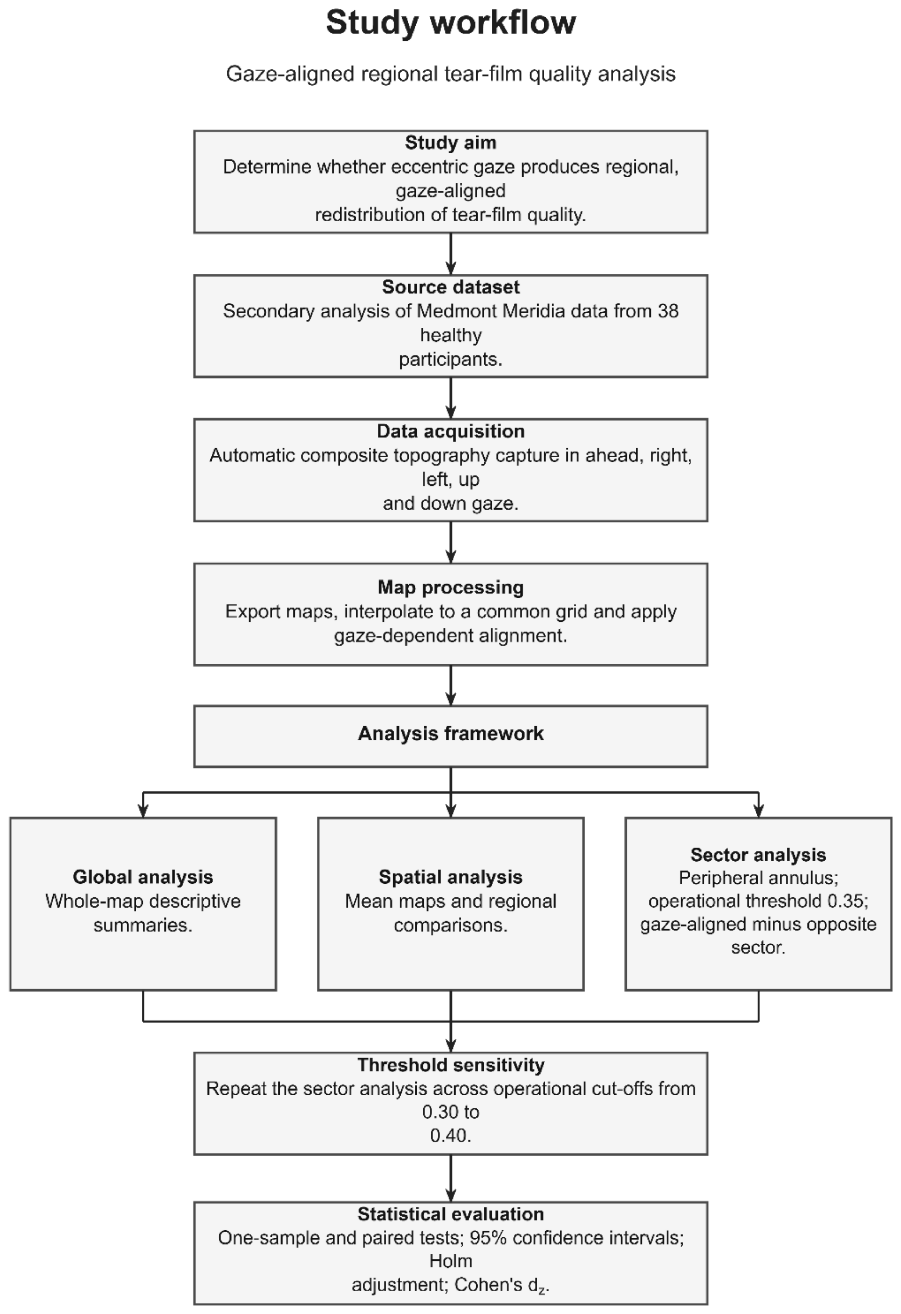
Workflow for five-gaze map alignment, regional and sector analysis, threshold sensitivity, and subject-level statistics in 38 healthy participants.

The methodological workflow, from the source dataset and map processing to spatial analyses, threshold-sensitivity testing and statistical evaluation, is summarised in Figure 1.

## 3. Results

### 3.1. Whole-Map and Spatial Findings

The analysed cohort comprised 38 healthy participants. The mean age was 36.5 ± 9.5 years (range, 24–60 years); 23 participants (60.5%) were female and 15 (39.5%) were male.

**Figure 2:**
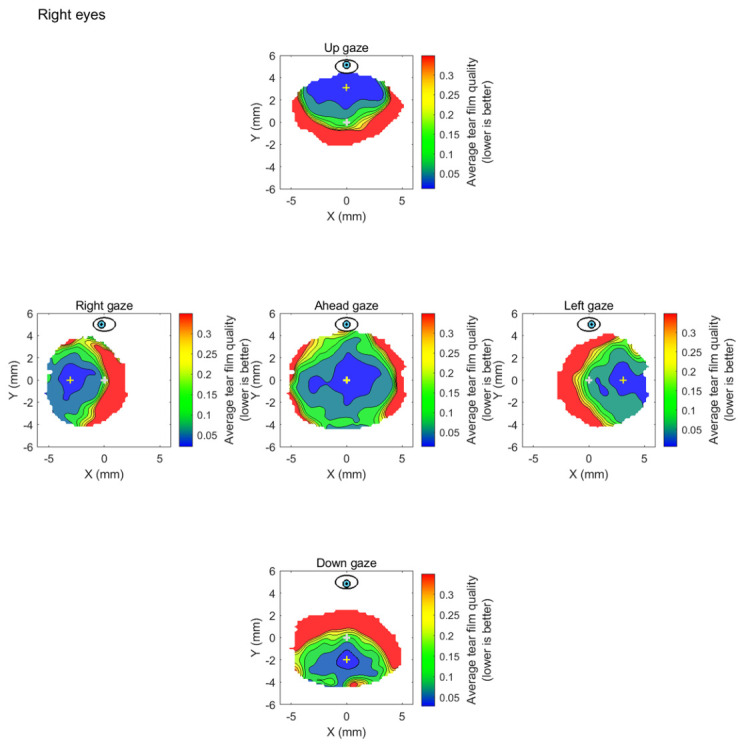
Participant-averaged tear-film quality maps for right eyes (*n* = 38) for ahead, right, left, up, and down gaze. Axes show map coordinates in millimetres, and the common colour scale represents the device-derived tear-film quality factor; lower values indicate better quality. Eccentric gaze produced localised redistribution rather than a uniform whole-map change.

**Figure 3:**
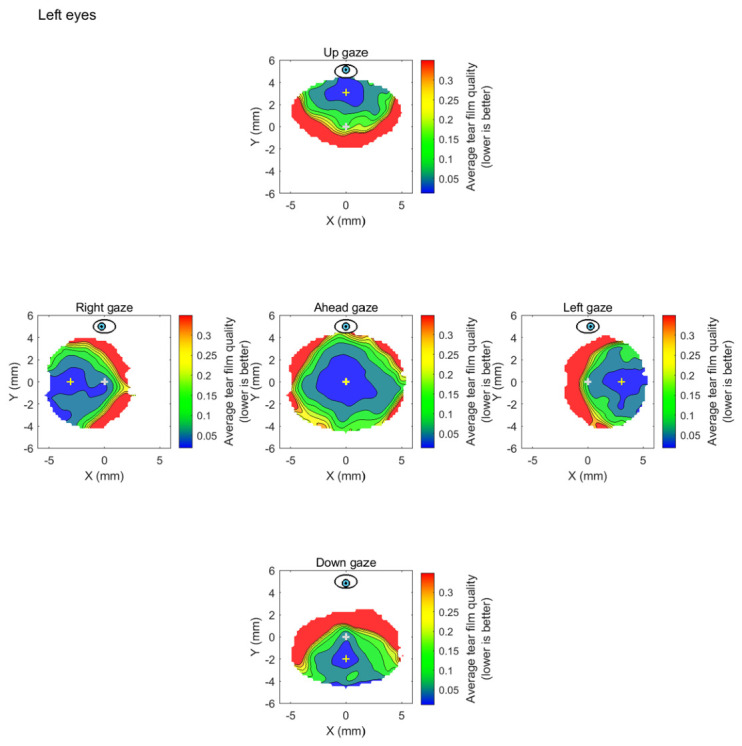
Participant-averaged tear-film quality maps for left eyes (*n* = 38) for ahead, right, left, up, and down gaze. Axes show map coordinates in millimetres, and the common colour scale represents the device-derived tear-film quality factor; lower values indicate better quality. Eccentric gaze produced localised redistribution rather than a uniform whole-map change.

**Figure 4:**
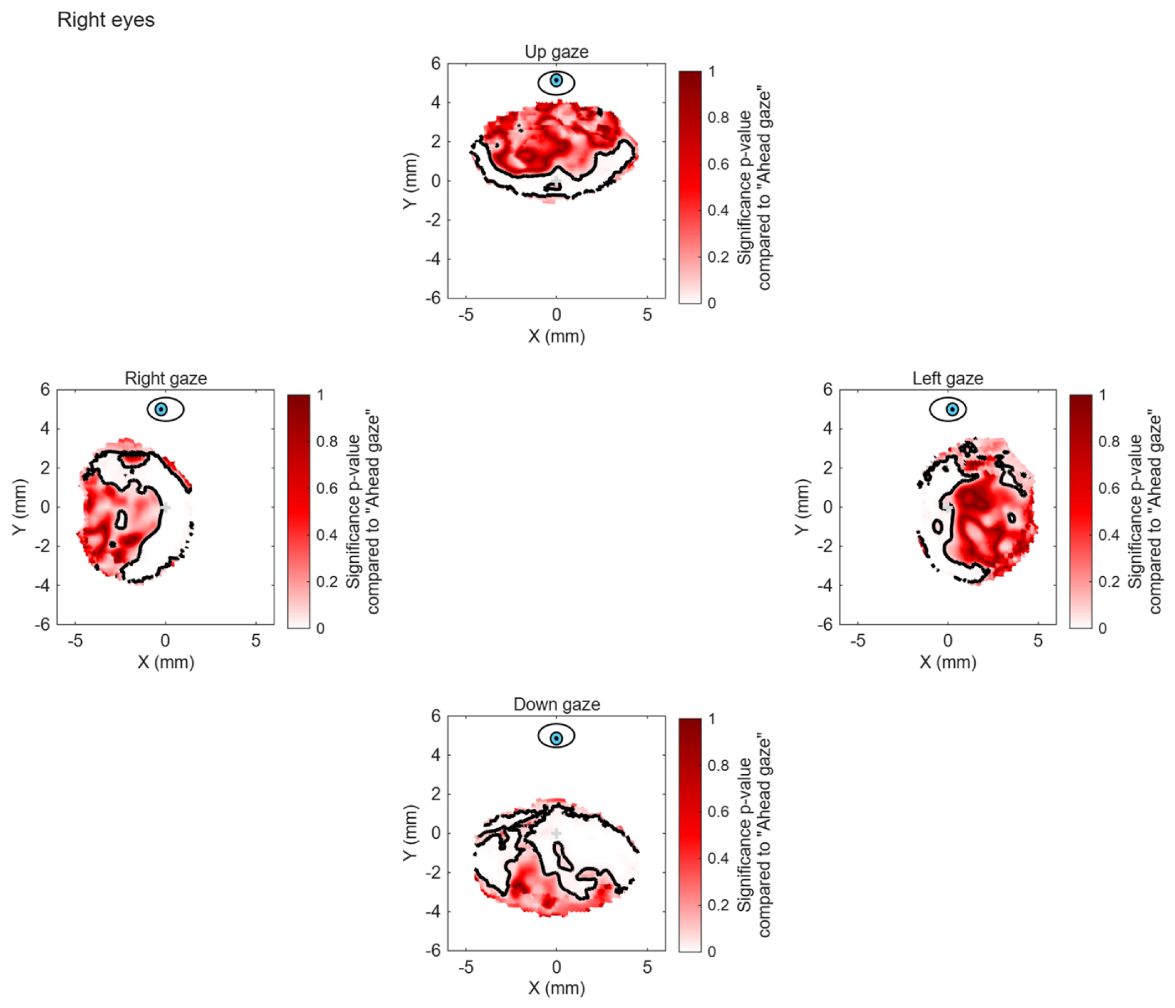
Descriptive regional comparison maps for right eyes (*n* = 38), with each eccentric gaze compared pointwise with ahead gaze. Darker shading indicates stronger evidence of a local difference, and black contours mark regions with uncorrected *p* < 0.05. The maps visualise spatial localisation and were not treated as multiplicity-corrected confirmatory tests.

**Figure 5:**
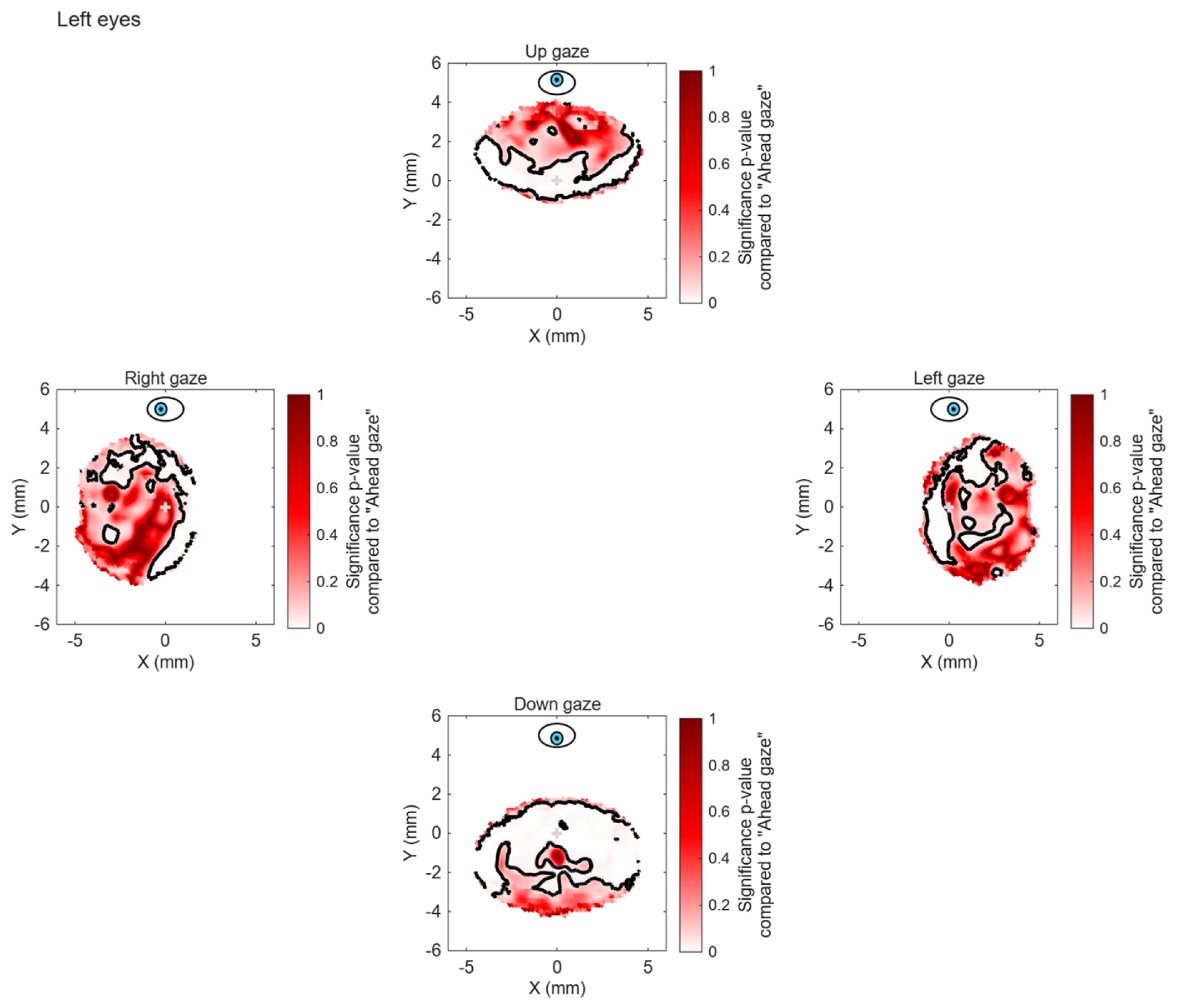
Descriptive regional comparison maps for left eyes (*n* = 38), with each eccentric gaze compared pointwise with ahead gaze. Darker shading indicates stronger evidence of a local difference, and black contours mark regions with uncorrected *p* < 0.05. The maps visualise spatial localisation and were not treated as multiplicity-corrected confirmatory tests.

Whole-map summary measures showed only limited variation with gaze direction. When the maps were reduced to a single overall value, the effect of gaze appeared modest, but this did not reflect the spatial appearance of the averaged maps. The mean tear-film quality maps showed that eccentric gaze redistributed relatively better- and poorer-quality regions locally rather than producing a uniform whole-map shift. These spatial patterns are shown separately for the right and left eyes in Figure 2 and Figure 3, and the corresponding regional comparison maps relative to ahead gaze are shown in Figure 4 and Figure 5.

### 3.2. Threshold-Based Sector Analysis

The threshold-based sector analysis provided a quantitative test of this regional pattern (Table 2). In the participant-level analysis, the proportion of good-quality pixels was higher in the gaze-aligned sector than in the opposite sector for all four eccentric gaze directions. The gaze-aligned good-pixel index was positive for right gaze (0.297 ± 0.200; 95% CI, 0.231 to 0.363; t(37) = 9.151; *p* < 0.001), left gaze (0.213 ± 0.174; 95% CI, 0.156 to 0.270; t(37) = 7.529; *p* < 0.001), up gaze (0.204 ± 0.219; 95% CI, 0.132 to 0.276; t(37) = 5.759; *p* < 0.001), and down gaze (0.308 ± 0.260; 95% CI, 0.221 to 0.395; t(37) = 7.213; *p* < 0.001). After Holm correction, all four indices remained significantly greater than zero (all pHolm < 0.001), indicating consistent gaze-aligned redistribution of threshold-defined better tear-film quality.

The same pattern was present when eccentric-gaze indices were compared with matched ahead-gaze sector indices. The mean eccentric-minus-ahead difference was 0.255 for right gaze (95% CI, 0.187 to 0.323; t(37) = 7.62; *p* < 0.001), 0.170 for left gaze (95% CI, 0.119 to 0.220; t(37) = 6.84; *p* < 0.001), 0.159 for up gaze (95% CI, 0.105 to 0.214; t(37) = 5.97; *p* < 0.001), and 0.264 for down gaze (95% CI, 0.181 to 0.347; t(37) = 6.46; *p* < 0.001). All four comparisons remained significant after Holm correction (all pHolm < 0.001). Thus, the directional pattern in eccentric gaze exceeded the corresponding ahead-gaze sector pattern and was not simply an artefact of sector geometry.

**Figure 6:**
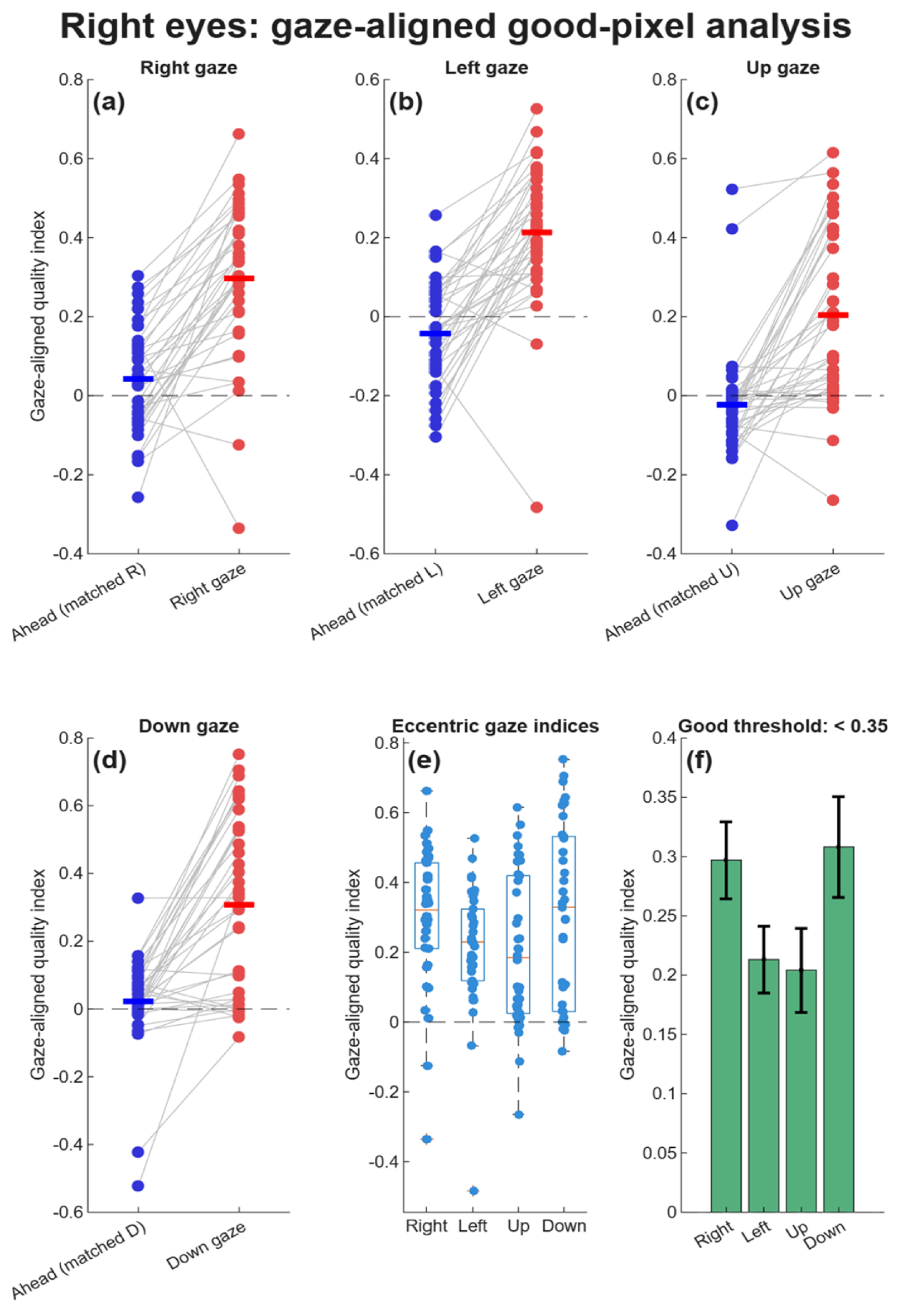
Right-eye threshold-based sector analysis (*n* = 38). Panels (**a**–**d**) compare matched ahead- and eccentric-gaze indices; grey lines connect participant pairs and horizontal bars show group means. Panel (**e**) shows participant-level eccentric-gaze indices, and panel (**f**) shows mean ± SEM. The index is the proportion of pixels below 0.35 in the gaze-aligned sector minus the opposite sector; positive values favour the gaze direction, and dashed lines mark zero. In panels (**a**–**d**), blue points represent matched ahead-gaze values and red points represent eccentric-gaze values; panel (**e**) shows individual eccentric-gaze indices in blue, and panel (**f**) shows group means ± SEM in green.

**Figure 7:**
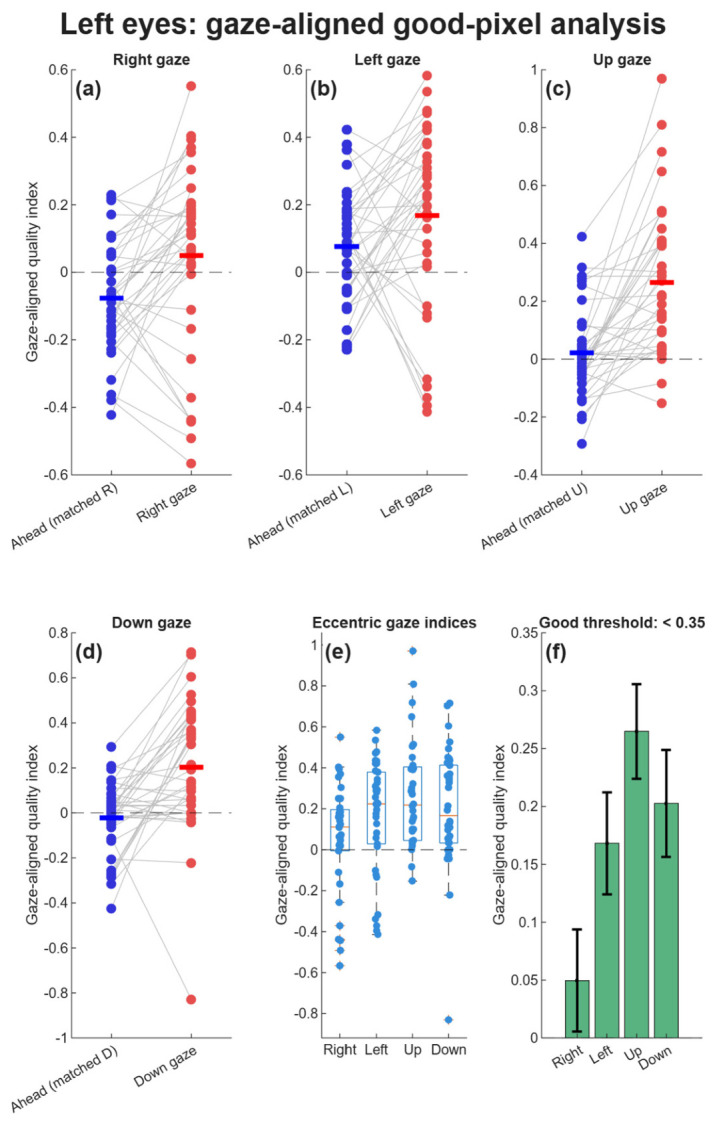
Left-eye threshold-based sector analysis (*n* = 38). Panels (**a**–**d**) compare matched ahead- and eccentric-gaze indices; grey lines connect participant pairs and horizontal bars show group means. Panel (**e**) shows participant-level eccentric-gaze indices, and panel (**f**) shows mean ± SEM. The index is the proportion of pixels below 0.35 in the gaze-aligned sector minus the opposite sector; positive values favour the gaze direction, and dashed lines mark zero. In panels (**a**–**d**), blue points represent matched ahead-gaze values and red points represent eccentric-gaze values; panel (**e**) shows individual eccentric-gaze indices in blue, and panel (**f**) shows group means ± SEM in green.

The subject-level paired plots and group summaries for these comparisons are shown for right and left eyes in Figure 6 and Figure 7, respectively.

### 3.3. Pooled Horizontal and Vertical Summaries

When right and left gaze were averaged to form a pooled horizontal index, the mean value was 0.255 ± 0.168 (95% CI, 0.200 to 0.310; t(37) = 9.37; *p* < 0.001). When up and down gaze were averaged to form a pooled vertical index, the mean value was 0.253 ± 0.169 (95% CI, 0.198 to 0.309; t(37) = 9.23; *p* < 0.001). The horizontal-minus-vertical difference was small and not statistically significant (mean difference, 0.002; 95% CI, −0.061 to 0.065; t(37) = 0.06; *p* = 0.954). The pooled results therefore support a robust gaze-aligned redistribution pattern but do not indicate a meaningful difference in overall effect size between the horizontal and vertical axes.

### 3.4. Threshold Sensitivity Analysis

Threshold sensitivity analysis confirmed that the main directional finding was not dependent on the single 0.35 cut-off (Table 3). Across thresholds from 0.30 to 0.40, the mean gaze-aligned index remained positive for all gaze directions: right gaze ranged from 0.285 to 0.306, left gaze from 0.194 to 0.234, up gaze from 0.194 to 0.217, and down gaze from 0.299 to 0.324. The largest p-value across this threshold range remained below 0.001 for each gaze direction. The pooled horizontal index remained positive across the same range (0.239 to 0.270), as did the pooled vertical index (0.243 to 0.267). The horizontal-versus-vertical comparison remained non-significant across the tested range (*p* = 0.856–0.997), reinforcing the fact that the most robust signal was a gaze-aligned regional redistribution rather than a large difference between the pooled axes. This indicates that the direction of the effect was stable and not driven by the choice of a single operational threshold. The stability of these findings across tested cut-offs is summarised in Table 3. The sensitivity analysis therefore strengthens the use of 0.35 as the primary operational threshold by showing that the main directional conclusion was unchanged across nearby cut-offs.

### 3.5. Summary of Findings

The study results show that tear-film quality changes with gaze direction in a regional and directionally organised manner. The effect is more evident in spatial maps and threshold-based sector indices than in global tear metrics alone. The 0.35 threshold analysis showed large, statistically robust gaze-aligned effects, and the threshold sensitivity analysis showed that these effects persisted across neighbouring cut-offs.

## 4. Discussion

The main contribution of this study is not the broad suggestion that gaze influences tear-film behaviour, as earlier work already points in that direction [8,9,10]. The more specific contribution is that the present analysis suggests that gaze-related change is regional, spatially organised, and consistently gaze-aligned across multiple gaze directions. In other words, the principal effect of gaze was not a large whole-map shift in average tear quality, but a stable redistribution of regional tear quality.

This interpretation fits comfortably within the broader tear-film framework. TFOS DEWS II places tear instability at the centre of dry-eye pathophysiology and emphasises the importance of tear behaviour on the exposed ocular surface [1,3,7]. The present study extends that concept by suggesting that the regional response to gaze is more structured than a simple global shift in tear quality. The pattern appears to vary across gaze directions, although the pooled horizontal-versus-vertical comparison did not show a clear difference in overall magnitude in the threshold-based analysis.

The gaze-aligned regional pattern distinguishes the present analysis from earlier studies. Pansell et al. showed that vertical gaze alters tear-film stability [8], and Čović et al. linked greater palpebral fissure height in primary gaze with poorer tear stability [9]. Ax et al. subsequently demonstrated movement of the tear film over regions newly exposed during vertical gaze change [10]. The present study complements these observations by quantifying a gaze-aligned regional pattern across four eccentric directions and by showing that the pattern persisted across neighbouring operational thresholds. Taken together, the evidence suggests that gaze-related tear behaviour involves organised redistribution across the exposed surface rather than only a uniform change in whole-map quality.

This interpretation is also compatible with the wider literature on tear evaporation, lipid-layer behaviour, meniscus dynamics, and exposure geometry. Experimental and clinical studies have shown that tear evaporation increases with greater exposed ocular-surface area, that lipid-layer integrity modulates evaporation resistance, and that tear menisci act as dynamic reservoirs that influence how tears are redistributed after each blink [22,23,26,27,28,29,30,31,34,46]. The same literature also suggests that incomplete blinking, prolonged interblink intervals, and altered task-related visual behaviour can modify local tear thinning and ocular-surface stress [11,12,13,14,15,16,33,35,36,37,38]. Based on this context, it is plausible that eccentric gaze changes are expressed regionally as gaze alters not only eye orientation, but also lid position, exposure geometry, and blink-mediated tear redistribution.

### 4.1. Potential Clinical Relevance and Future Research

Current dry-eye frameworks place tear-film instability at the centre of ocular-surface assessment [1,6]. However, as this study included healthy participants and did not assess symptoms, clinical outcomes or diagnostic performance, the findings do not establish a clinical application. They show only that tear-film quality can vary regionally with gaze and that primary-gaze measurements may not fully describe behaviour during eccentric viewing.

Future studies should test whether this pattern is altered in symptomatic dry-eye populations, whether it relates to task-specific symptoms, and whether it adds information beyond conventional measurements during reading, screen use or other sustained visual tasks [16,50]. Multi-gaze mapping should therefore be regarded as a framework for clinical validation rather than a recommended diagnostic test.

### 4.2. Limitations

This study has several limitations. First, the tear-film quality factor is a mapped, device-derived variable rather than a conventional clinical endpoint such as non-invasive or fluorescein tear break-up time. The measure was appropriate for evaluating spatial redistribution, but it cannot be interpreted directly as tear stability time or diagnostic performance [51]. Second, the sample was modest and drawn from a single existing cohort of healthy participants. The sensitivity analysis indicated adequate power for moderate within-subject effects, but the study was not designed for age-, sex-, or disease-subgroup analyses. Third, the secondary design meant that participant selection, acquisition procedures, and image acceptability were inherited from the source dataset, creating potential selection and acquisition biases that could not be examined prospectively. Fourth, the static gaze maps do not capture the full time-dependent effects of blinking, eye movements, or sustained visual tasks. Finally, the results may not generalise to older populations, symptomatic dry-eye groups, or other imaging platforms. These questions require prospective, multi-centre validation.

Despite these limitations, the consistency of the findings across gaze directions and operational thresholds supports the presence of a measurable regional tear-film signal in this healthy cohort.

## 5. Conclusions

Tear-film quality changed with gaze direction in a regional and directionally organised manner. Spatial mapping and threshold-based sector analysis revealed this pattern more clearly than whole-map measures. The consistently positive gaze-aligned indices indicate that threshold-defined better-quality regions tended to align with the direction of gaze. These findings establish a physiological pattern in healthy eyes and provide a basis for future studies in symptomatic populations and during functional visual tasks. Clinical utility and diagnostic performance remain to be determined.

## Figures and Tables

**Table 2:** Threshold-based gaze-aligned good-pixel sector analysis at the 0.35 operational threshold.

Gaze	Good Pixels: Aligned Sector	Good Pixels: Opposite Sector	Index, Mean ± SD	95% CI	t (df)	pHolm vs. 0	Cohen dz
Right	0.889	0.592	0.297 ± 0.200	0.231 to 0.363	9.151 (37)	<0.001	1.484
Left	0.914	0.700	0.213 ± 0.174	0.156 to 0.270	7.529 (37)	<0.001	1.221
Up	0.942	0.738	0.204 ± 0.219	0.132 to 0.276	5.759 (37)	<0.001	0.934
Down	0.875	0.567	0.308 ± 0.260	0.221 to 0.395	7.213 (37)	<0.001	1.186

Note: The index is the proportion of good-quality pixels in the gaze-aligned sector minus the proportion in the opposite sector. Positive values indicate there is a greater number of good-quality pixels towards the direction of gaze. All rows include *n* = 38 participants and 37 degrees of freedom. SD, standard deviation; CI, confidence interval; dz, standardised within-subject effect size. pHolm values are two-sided one-sample p-values for testing the index against zero, adjusted across the four gaze-direction comparisons using the Holm method.

**Table 3:** Threshold sensitivity analysis across operational cut-offs from 0.30 to 0.40 (*n* = 38).

Measure	Mean Index Range	Largest *p*-Value	Interpretation
Right	0.285 to 0.306	<0.001	Positive and significant across tested thresholds
Left	0.194 to 0.234	<0.001	
Up	0.194 to 0.217	<0.001	
Down	0.299 to 0.324	<0.001	
Horizontal pooled	0.239 to 0.270	<0.001	
Vertical pooled	0.243 to 0.267	<0.001	

Note: Each index range is the minimum-to-maximum group mean observed across operational cut-offs from 0.30 to 0.40. The largest *p*-value is the largest two-sided one-sample p-value for testing the relevant mean index against zero across those cut-offs. All analyses included *n* = 38 participants.

## Data Availability

Related data analyses of the source Medmont dataset have been reported previously [39]. Additional access to the underlying data and the analysis code will be considered by the corresponding author on reasonable request.
